# Poor Identification of Emergency Department Acute Recreational Drug Toxicity Presentations Using Routine Hospital Coding Systems: the Experience in Denmark, Switzerland and the UK

**DOI:** 10.1007/s13181-018-0687-z

**Published:** 2019-01-02

**Authors:** David M. Wood, Luke De La Rue, Ali A. Hosin, Gesche Jurgens, Evangelia Liakoni, Fritdjof Heyerdahl, Knut Erik Hovda, Alison Dines, Isabelle Giraudon, Matthias E. Liechti, Paul I. Dargan

**Affiliations:** 1 Clinical Toxicology, Guy’s and St Thomas’ NHS Foundation Trust and King’s Health Partners, St Thomas’ Hospital, 3rd Floor, Block C, South Wing, Westminster Bridge Road, London, SE1 7EH UK; 2 Faculty of Life Sciences and Medicine, King’s College London, Stamford St, Lambeth, London, SE1 9NH UK; 3 General Medicine, University College Hospital London, 235 Euston Rd, Fitzrovia, London, NW1 2BU UK; 4 Clinical Pharmacology Unit, Zealand University Hospital, Sygehusvej 10, 4000 Roskilde, Denmark; 5 Division of Clinical Pharmacology and Toxicology, Basel University Hospital, Spitalstrasse 21, 4031 Basel, Switzerland; 6 University of Basel, Petersplatz 1, 4001 Basel, Switzerland; 7 The Norwegian CBRNE Centre of Medicine, Department of Acute Medicine, Ullevål Hospital, Kirkeveien 166, 0450 Oslo, Norway; 8 Risks to public safety and security unit, European Monitoring Centre for Drugs and Drug Addiction (EMCDDA), Praça Europa 1, Cais do Sodré, 1249-289 Lisbon, Portugal

**Keywords:** Recreational drugs, Novel psychoactive substances, Acute toxicity, Clinical coding, Emergency department

## Abstract

**Background:**

Understanding emergency department and healthcare utilisation related to acute recreational drug toxicity (ARDT) generally relies on nationally collated data based on ICD-10 coding. Previous UK studies have shown this poorly captures the true ARDT burden. The aim of this study was to investigate whether this is also the case elsewhere in Europe.

**Methods:**

The Euro-DEN Plus database was interrogated for all presentations 1st July to 31st December 2015 to the EDs in (i) St Thomas’ Hospital, London, UK; (ii) Universitätsspital Basel, Basel, Switzerland; and (iii) Zealand University Hospital, Roskilde, Denmark. Comparison of the drug(s) involved in the presentation with the ICD-10 codes applied to those presentations was undertaken to determine the proportion of cases where the primary/subsequent ICD-10 code(s) were ARDT related.

**Results:**

There were 619 presentations over the 6-month period. Two hundred thirteen (34.4%) of those presentations were coded; 89.7% had a primary/subsequent ARDT-related ICD-10 code. One hundred percent of presentations to Roskilde had a primary ARDT ICD-10 code compared to 9.6% and 18.9% in Basel and London respectively. Overall, only 8.5% of the coded presentations had codes that captured all of the drugs that were involved in that presentation.

**Conclusions:**

While the majority of primary and secondary codes applied related to ARDT, often they did not identify the actual drug(s) involved. This was due to both inconsistencies in the ICD‐10 codes applied and lack of ICD‐10 codes for the drugs/NPS. Further work and education is needed to improve consistency of use of current ICD‐10 and future potential ICD‐11 coding systems.

## Introduction

The International Classification of Disease version 10 (ICD-10) system is often used to code hospital outpatient attendances, emergency department (ED) attendances and hospital discharges and used to understand the burden on healthcare systems related to specific diseases/medical conditions [1]. The Hospital Episode Statistics (HES) in the UK collate over 125 million ICD-10 coded episodes per year and HES data is used to demonstrate the prevalence of different medical conditions across the UK, as well as for planning and funding health service provision [1].

Although ICD-10 coding can generate representative data for routine surgical admissions to hospital and for general medical conditions (for example, cancer and heart disease), previous UK studies have shown it is less robust at capturing recreational drug-related burden of health care utilisation [2, 3]. There are a number of reasons that potentially explain this. First, in many countries in Europe, ICD-10 codes are usually only applied to patients admitted to hospital beyond the ED, based on local country coding mechanisms. Over three quarters of acute recreational drug toxicity (ARDT) patients are discharged directly from the ED, and since they are not admitted to hospital, they are not captured by the ICD-10 coding system [4]. ED presentations not admitted to hospital may be coded at a “high level” of ICD-10 codes, rather than by the detailed sub-classifications of ICD-10 codes leading to less granularity of coding for the episode [1–3]. Second, many established recreational drugs (e.g. MDMA, ketamine) lack formal ICD-10 codes and therefore coding of those substances specifically is not possible. Similarly, the rapidly emerging number of new psychoactive substances (NPS) is also not codeable under existing ICD-10 codes [1–3]. Finally, national coding collation systems, such as HES, typically report on primary ICD-10 diagnostic code, and even where codes do exist, the primary diagnostic code applied to an admission may reflect the condition caused by the recreational drug rather than the recreational drug used. For example, cocaine-related myocardial infarction may have a primary code of myocardial infarction with cocaine coded as a secondary or subsequent diagnostic code. Using only the primary ICD-10 code will mean the recreational drug involvement will not be captured.

Therefore, nationally collated ICD-10 coded data in the UK means that the true burden of ARDT-related health care utilisation is not understood [1–6]. However, there have been no studies to determine whether these issues are only representative of the UK, or whether this also occurs in other European healthcare systems. In this study, we have utilised known ED presentations with acute recreational drug and NPS toxicity reported to the European Drug Emergencies Network Plus (Euro-DEN Plus) project from three cities in three different European countries (Denmark, Switzerland, and the UK) to further understand the issues of using ICD-10 coded data to capture the healthcare burden related to their use and we sought to determine if there is concordance between ICD-10 coding and Euro-DEN Plus case coding of recreational drugs and NPS used in three distinct European medical centres.

## Methods

The development of the initial Euro-DEN project and the local approval processes have been previously described [4, 7]. Briefly, the initial Euro-DEN project and the subsequent Euro-DEN Plus projects collect the following data from routine medical notes of all patients presenting with acute recreational drug (including NPS) toxicity: patient demographics, drug(s) used, clinical features associated with the presentation and physiological observations on presentation, treatment(s) given, disposition from ED (and overall survival to discharge), and length of stay [4, 7, 8]. Each Euro-DEN Plus centre is responsible for ensuring all relevant cases are identified and entered into the project database. The Euro-DEN Plus database was interrogated to identify all presentations between 1st July 2015 and 31st December 2015 to the three Euro-DEN Plus centres participating in this study: (i) St Thomas’ Hospital, London, UK; (ii) the University Hospital of Basel (Universitätsspital Basel), Basel, Switzerland; and (iii) Zealand University Hospital, Roskilde, Denmark. For each presentation, the reported drug(s) used were extracted. All of these hospitals are urban hospitals co-located with universities and act both as secondary care providers for their local residents and are tertiary referral centres for specialist care.

The central hospital coding departments at participating hospitals were asked in the first quarter of 2017 whether any ICD-10 codes had been applied to each presentation and what codes had been applied in order of coding (primary, secondary and subsequent codes). The coding had been undertaken part of routine clinical care in each hospital at the time of the presentation. Those undertaking the coding were not aware of this future study.

Analysis was undertaken to determine (i) the proportion of cases where there were no diagnostic codes recorded at all, (ii) the proportion of cases where the primary diagnostic codes included an ARDT-related code (defined as one of the following ICD-10 codes: F11–F19, T40–43, T50, T59 or T65), (iii) the proportion of cases where a second and/or subsequent ARDT-related code was recorded (defined as one of the following ICD-10 codes: F11–F19, T40–43, T50, T59 or T65 along with X40, X41, X42, X44, X47, X61, X62, X64 and Z27.2), and (iv) the most common types of primary coding recorded for those presentations where no code related to ARDT was recorded. In addition, analysis was undertaken to determine whether ICD-10 codes related to the drug category(ies) used (defined as stimulants, cannabinoids, hallucinogens and depressants). Finally, sub-analysis comparing the concordance of drugs reported in the Euro-DEN Plus cases to the ICD-10-coded drugs was undertaken for the opioids, cannabinoids, cocaine, and mephedrone. These drugs were chosen due to the frequency of use, concerns about harms related to their use and/or that there have specific codes in the ICD-10 coding system that identify them.

### Statistical Analysis

Descriptive statistics, undertaken using Excel 2013, were used to describe the proportion of cases in each group during any analysis.

## Results

**Fig. 1:**
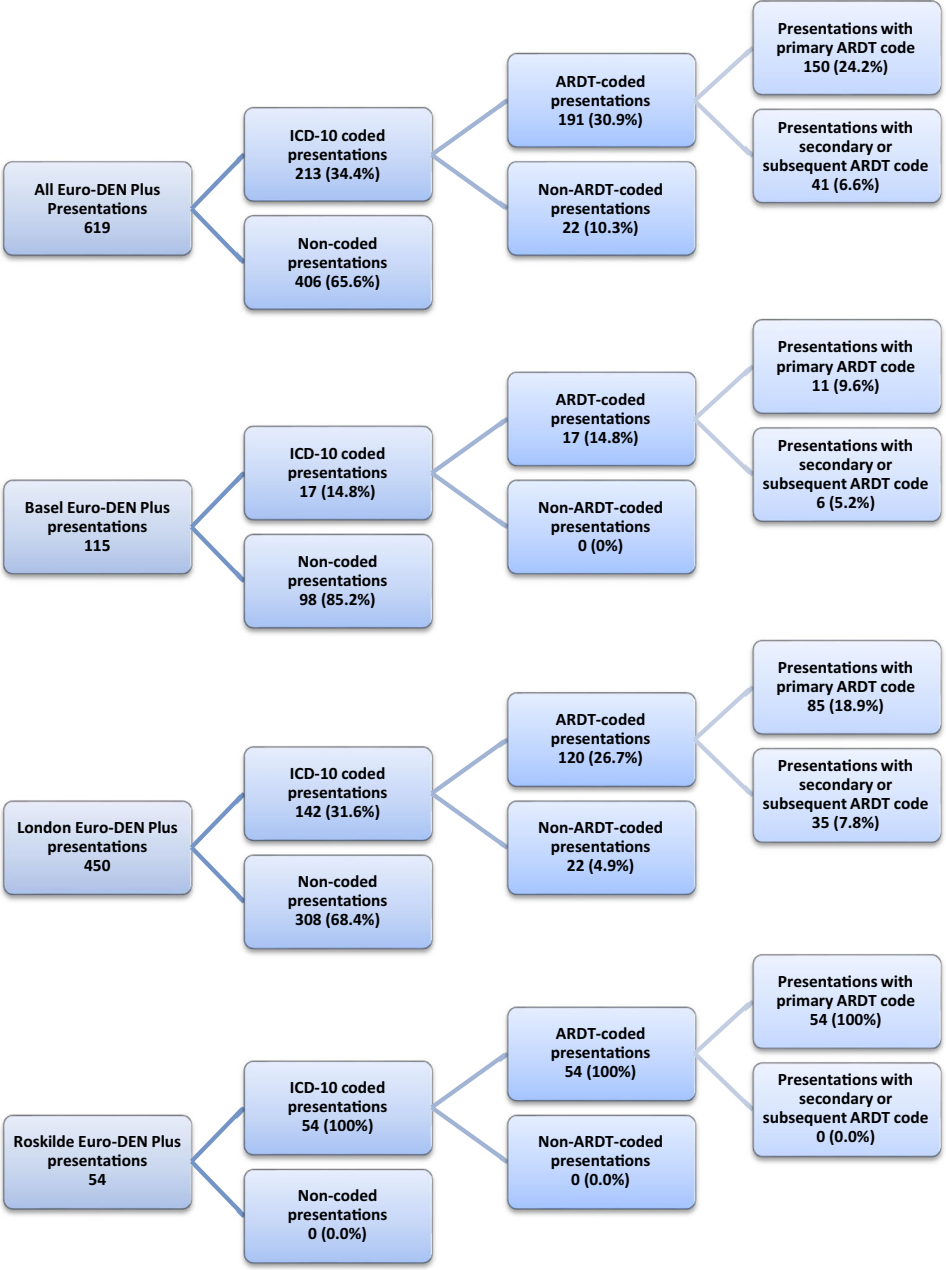
Summary of coding and application of acute recreational drug-related ICD-10 codes for all cases and by centre involved in the study

During the 6-month study period, there were a total of 619 ARDT-related presentations to the three participating Euro-DEN Plus centres (London, 450; Basel, 115; Roskilde, 54). Of these presentations, 213 (34.4%) had one or more ICD-10 codes applied to that presentation, with variability between the centres: Basel, 14.8%; London, 31.6% and Roskilde, 100% (Fig. 1). Four hundred six (71.9%) of London and Basel presentations did not have any ICD-10 codes applied; in 365 (89.9% of uncoded episodes), the patient was not admitted beyond the ED (Basel, 95; London, 270): (i) medically discharged 302 (219 London; 83 Basel), (ii) self-discharged and/or did not wait 62 (50 London; 12 Basel) or (iii) died in the ED 1 (London).

### ICD-10 Clinical Coding

Of the 213 coded presentations, 191 (89.7% of coded presentations) had either a primary, secondary and/or subsequent ARDT (ARDT) code. Twenty-two coded presentations had no ARDT-related ICD-10 codes applied; all of these were in London.

#### Primary Clinical Code

One hundred fifty of the presentations (24.2% of all presentations, 70.4% of coded presentations) had a primary ARDT ICD-10 code. All of the Roskilde presentations had a primary ARDT code, compared to 11 (9.6% of total presentations) in Basel and 85 (18.9%) in London (Fig. 1). Overall, there were 27 different primary ICD-10 codes applied, of which 7 (71 presentations) related to “mental and behavioural disorders” group of codes (F codes) and 20 (79 presentations) related to “poisoning/toxic effects” group of codes (T codes) (Table 1). The 63 presentations without a primary ARDT-related ICD-10 code had 36 different primary ICD-10 codes applied (Table 2); the most commonly used codes related to: (i) alcohol (15 presentations), (ii) mental health (15 presentations) and (iii) head injury (5 presentations). Of the 15 coded with an alcohol-related ICD-10 code, all had recorded whether alcohol had either been used (13 (86.7%)) or not used (2 (13.3%)).

**Table 1:** Frequency of specific primary (150 presentations) and subsequent (41 presentations) ICD-10 diagnostic codes related to acute recreational drug toxicity (N/A - not applicable as X codes relate to intention of exposure and cannot be primary ICD-10 code applied)

ICD-10 code	Descriptive ICD-10 code	Primary code	Secondary/subsequent
		(*n*)	(*n*)
F11	Mental and behavioural disorders due to use of opioids	13	17
F12	Mental and behavioural disorders due to use of cannabinoids	13	12
F13	Mental and behavioural disorders due to use of sedatives or hypnotics	5	18
F14	Mental and behavioural disorders due to use of cocaine	9	23
F15	Mental and behavioural disorders due to use of other stimulants	12	27
F16	Mental and behavioural disorders due to use of hallucinogens	3	0
F17	Mental and behavioural disorders due to use of tobacco	0	19
F18	Mental and behavioural disorders due to use of volatile solvents	0	1
F19	Mental and behavioural disorders due to multiple drug use of other psychoactive substances	16	6
T40	Poisoning by narcotics and psychodysleptics (hallucinogens)		
T40.1	Heroin	6	4
T40.2	Other opioids	1	1
T40.3	Methadone	5	2
T40.4	Other synthetic narcotics	1	0
T40.5	Cocaine	10	3
T40.6	Other and unspecified narcotics	5	0
T40.7	Cannabis	4	2
T40.8	Lysergide (LSD)	2	0
T40.9	Other and unspecified psychodysleptics (hallucinogens)	2	0
T41	Poisoning by anaesthetics and therapeutic gases		
T41.0	Intravenous anaesthetics	1	0
T41.2	Other and unspecified general anaesthetics	9	2
T42	Poisoning by antiepileptic, sedative-hypnotic and antiparkinsonism drugs		
T42.4	Benzodiazepines	1	3
T42.6	Other antiepileptic and sedative-hypnotic drugs	1	1
T43	Poisoning by psychotropic drugs, not elsewhere classified		
T43.0	Tricyclic and tetracyclic antidepressants	1	0
T43.2	Other and unspecified antidepressants	0	1
T43.5	Other and unspecified antipsychotics and neuroleptics	2	0
T43.6	Psychostimulants with abuse potential (excl. cocaine)	17	7
T43.8	Other psychotropic drugs, not elsewhere specified	5	1
T46	Poisoning by agents primarily affecting the cardiovascular system		
T46.3	Coronary vasodilators, not elsewhere classified	0	1
T46.7	Peripheral vasodilators	0	1
T50	Poisoning by diuretics and other and unspecified drugs, medicaments and biological substances		
T50.9	Poisoning: other and unspecified drugs, medicaments and biological substances	4	0
T59	Toxic effect of other gases, fumes and vapours		
T59.8	Other specified gases, fumes and vapours	1	0
T65	Toxic effect of other and unspecified substances		
T65.9	Toxic effect of unspecified substance	1	0
T88.7	Unspecified adverse effect of drug or medicament	0	1
X40	Accidental poisoning by and exposure to nonopioid analgesics, antipyretics and antirheumatics	N/A	1
X41	Accidental poisoning by and exposure to antiepileptic, sedative-hypnotic, antiparkinsonism and psychotropic drugs, not elsewhere classified	N/A	19
X42	Accidental poisoning by and exposure to narcotics and psychodysleptics (hallucinogens), not elsewhere specified	N/A	16
X44	Accidental poisoning by and exposure to other and unspecified drugs, medicaments and biological substances	N/A	15
X47	Accidental poisoning by and exposure to other gases and vapours	N/A	1
X61	Intentional self-poisoning by and exposure to antiepileptic, sedative-hypnotic, antiparkinsonism and psychotropic drugs, not elsewhere specified	N/A	9
X62	Intentional self-poisoning by and exposure to narcotics and psychodysleptics (hallucinogens), not elsewhere classified	N/A	7
X64	Intentional self-poisoning by and exposure to other and unspecified drugs, medicaments and biological substances	N/A	6
Z72.2	Drug use	0	1

**Table 2:** Primary ICD-10 diagnostic codes not related to acute recreational drug toxicity

ICD-10 code	Descriptive ICD-10 code	Cases applied to
		(*n*)
Metabolic/endocrine (6 presentations)		
D74.9	Methaemoglobinaemia, unspecified	2
E78.1	Pure hyperglyceridaemia	1
E87.2	Acidosis	1
T38.8	Other and unspecified hormones and their synthetic substitutes	1
N17.9	Acute renal failure, unspecified	1
Alcohol related (15 presentations)		
F10.0	Mental and behavioural disorders due to use of alcohol	11
F10.3	Mental and behavioural disorders due to use of alcohol (withdrawal state)	3
T51.0	Ethanol	1
Neuropsychiatric (21 presentations)		
F22.0	Delusional disorder	1
F29	Unspecified nonorganic psychosis	2
F41.0	Panic disorder (episodic paroxysmal anxiety)	3
F41.9	Anxiety disorder, unspecified	2
G40.2	Localization-related (focal) (partial) symptomatic epilepsy and epileptic syndromes with complex partial seizures	1
G41.0	Grand mal status epilepticus	1
G93.6	Cerebral oedema	1
R45.2	Unhappiness	1
R45.8	Other symptoms and signs involving emotional state (suicidal ideation (tendencies))	6
R55	Syncope and collapse	3
Cardiorespiratory (8 presentations)		
I26.9	Pulmonary embolism without mention of acute cor pulmonale	1
I46.0	Cardiac arrest with successful resuscitation	1
J14	Pneumonia due to *Haemophilus influenzae*	1
J69.0	Pneumonitis due to food and vomit	2
R06.8	Other and unspecified abnormalities of breathing	1
R07.4	Chest pain unspecified	2
Gastrointestinal (3 presentations)		
K92.2	Gastrointestinal haemorrhage unspecified	1
R10.4	Other and unspecified abdominal pain	1
R11	Nausea and vomiting	1
Trauma/injury related (8 presentations)		
S00.7	Multiple superficial injuries of the head	1
S00.8	Superficial injury of other parts of the head	1
S01.0	Open wound of scalp	1
S01.1	Open wound of eyelid and periocular area	1
S09.9	Unspecified injury of the head	2
T00.8	Superficial injuries involving other combinations of body regions	1
T14.2	Fracture of unspecified body region	1
Other (2 presentations)		
M46.4	Discitis, unspecified	1
M54.2	Cervicalgia	1

#### Secondary or Subsequent Clinical Codes

A further 41 presentations (6.6% of all presentations, 19.2% of coded presentations) would be identified as a recreational drug-related acute toxicity presentation from a secondary or subsequent ICD-10 ARDT codes. There were 31 different ICD-10 codes applied as secondary/subsequent codes, of which 8 related to the “mental and behavioural disorders” group of codes (F codes), 14 related to “poisoning/toxic effects” group of codes (T codes), 9 related to the “external causes of morbidity and mortality” and/or “factors influencing health status” groups of codes (X and Z codes) (Table 1).

### Concordance of Euro-DEN Plus Drugs Identified and Drugs Identified by ICD-10 Clinical Codes

Of the 213 ICD-10-coded presentations, only 18 (8.5%) had ICD-10 codes applied which captured all of the recreational drug(s) that were involved in an individual Euro-DEN Plus presentation (for example, if they had used cannabis and cocaine and ICD-10 codes for cannabis and cocaine had been applied), ranging from 0.7 in London to 29.6% in Roskilde (Table 3); in addition, a further 33 (15.5%) presentations had ICD-10 codes which captured at least one but not all of the drug(s) that had been reported in that individual presentation (for example, if they had used cannabis and cocaine but only an ICD-10 code for cannabis had been applied). This meant that a total of 162 (76.1%) presentations had ICD-10 codes for drug(s) other than those that had been recorded for that presentation in the Euro-DEN Plus database (for example, a patient who had used cannabis and cocaine but an ICD-10 code for heroin only was applied).

**Table 3:** Concordance of ICD-10 codes with drugs captured in each presentation by Euro-DEN by site

	Basel		London		Roskilde		Combined	
	No.	%	No.	%	No.	%	No.	%
ICD-10-coded presentations	17		142		54		213	
All drugs captured by Euro-DEN	1	5.9	1	0.7	16	29.6	18	8.5
At least one drug captured by Euro-DEN	6	35.3	26	18.3	19	35.2	51	23.9
No drugs captured by Euro-DEN	11	64.7	116	81.7	35	64.8	162	76.1

The accuracy of ICD-10 coding and the detection of presentations by searching applied ICD-10 codes for opioids, cannabinoids, cocaine and the NPS mephedrone is summarised below.

#### Opioids

- Twenty-five presentations had a primary opioid toxicity-related ICD-10 code (F11: mental and behaviour disorders due to use of opioids or T40.1, T40.2, T40.3: poisoning by heroin, other opioids or methadone respectively): 12 (48.0%) had reported use of an opioid.
- Ninety-six Euro-DEN Plus presentations included the use of an opioid: 8 (8.3%) would be identified by searching primary ICD-10 codes and 14 (14.6%) by subsequent ICD-10 codes equating to an overall detection rate of 22.9%.

#### Cocaine

- Nineteen presentations had a primary cocaine toxicity-related ICD-10 code (F14: mental and behaviour disorders due to use of cocaine or T40.5: poisoning by cocaine): 16 (84.2%) had reported use of cocaine.
- One hundred forty-nine Euro-DEN Plus presentations included the use of cocaine: 17 (11.4%) would be identified by searching primary ICD-10 codes and 12 (8.1%) by subsequent ICD-10 codes equating to an overall detection rate of 19.5%.

#### Cannabis and Synthetic Cannabinoids

- Seventeen presentations had a primary cannabinoid-related ICD-10 code (F12: mental and behaviour disorders due to use of cannabinoids or T40.7: poisoning by cannabis): 15 (88.2%) had reported use of cannabis and/or synthetic cannabinoids.
- One hundred sixteen Euro-DEN Plus presentations included the use of cannabis and/or synthetic cannabinoids: 15 (12.9%) would be identified by searching primary ICD-10 codes and 6 (5.2%) by subsequent ICD-10 codes equating to an overall detection rate of 18.1%.

#### Mephedrone

- There are no ICD-10 codes that relate directly to mephedrone.
- Forty-six Euro-DEN Plus presentations had used mephedrone: none would be identified directly by searching primary or subsequent ICD-10 codes. Twenty-one mephedrone presentations were coded: 12 (57.1%) had primary ICD-10 codes that were toxicity/poisoning ICD-10 codes (poisoning by non-specific stimulants (5), cocaine (2), general anaesthetics (2), alcohol (1), methadone (1) and cannabis (1)).

## Discussion

Approximately one third of European Drug Emergencies Network (Euro-DEN) Plus presentations to the EDs in the three cities (London, UK; Roskilde, Denmark and Basel, Switzerland) were coded using the ICD-10 coding systems, although there was variability in the proportion that were coded by centre. This study has demonstrated that the majority of primary and secondary codes applied related to acute recreational drug toxicity, but often they were not specific to the drug(s) used. The concordance of ICD-10 coding with drugs used showed high specificity but low sensitivity: those presentations coded with a specific drug ICD-10 code were likely to have taken that drug but the majority who had used the drug were not likely to be have the relevant ICD-10 code applied.

Previous studies have shown that around 50% of presentations with ARDT are discharged directly from the ED, and these presentations are unlikely to be coded using the ICD-10 coding system as this relates to patients admitted to hospital [2, 3]. In this study, in the two centres (Basel and London) with more uncoded Euro-DEN Plus presentations, nearly 90% of these had only been managed within the ED, consistent with those previously reported studies [4]. However, in Roskilde, Denmark, all of the presentations were coded as there is a requirement for all hospital presentations to be coded and reported to the National Register (Landspatientregisteret). Use of ICD-10 data in Denmark would potentially capture the burden of number of presentations; it would not allow true understanding of the burden for an individual drug, since the codes applied were not necessarily accurate for the drug(s) used. Further work is needed to understand whether other European countries utilise different methods for coding emergency presentations to see whether this variability in the capture of recreational drug presentations is replicated elsewhere.

Of the primary ICD-10 codes applied to the Euro-DEN Plus presentations, around 70% could be classified as a code that related to either the mental and behavioural or poisoning/toxicological effects of a recreational drug. Of the 213 presentations where a primary diagnostic code was recorded, only 150 (70.4%) received a primary ICD-10 diagnostic code that was related to ARDT. Some of the “acute drug toxicity”-related ICD-10 codes applied may not at initial review be consistent with the use of a recreational drug. One example is those presentations that had a primary ICD-10 code related to poisoning by anaesthetics agents; it is possible that some of these presentations related to the acute toxicity from either GBL/GHB or ketamine use since both GHB and ketamine have been used as anaesthetics and there is no specific ICD-10 code for these drugs. In a previous study, where clinical coders coded simulated ARDT presentations, the ICD-10 diagnostic code “T41.2 Poisoning by other and unspecified general anaesthetics” was used by 14% for a GBL-related collapse in a sauna [3]. The non-ARDT primary ICD-10 codes applied related to a range of other medical conditions, which could be considered as caused by the use of a recreational drugs/NPS such as acute mental health or neuropsychiatric disorders, cardiac toxicity or trauma. It is also interesting to note that the majority intent-related ICD-10 codes applied related to accidental rather than intentional poisoning. It is unknown whether coders used accidental codes as individuals are unlikely to use them with an intent to overdose, even if there is intentional use of a substance that could be associated with the risk of acute toxicity when used.

The main issue identified with this study is likely under detection of presentations through the utilisation of searching by ICD-10 codes. In a previous retrospective review of 484 known acute recreational toxicity presentations to an ED in London, UK, only 13% had been coded with a primary ICD-10 code that related to ARDT and overall looking at levels of coding, only approximately 20% of presentations had one or more ICD-10 codes applied that could relate to ARDT [2]. There was high specificity to detect specific drugs, since when an ICD-10 code was applied to a presentation, the majority of those presentations involved the use of that drug. However, there was low sensitivity since the majority of those who had actually used a specific drug did not have the relevant drug-specific ICD-10 code applied to the presentation. NPS will not have specific ICD-10 codes, since they have not been used for as long as the current ICD codes have been. Mephedrone was the most widely used and available NPS at the time of these Euro-DEN Plus presentations; since there is no ICD-10 code for mephedrone, it is not possible to look at coded presentations to detect mephedrone use. In part, this is due to ICD-10 having been endorsed for use by the World Health Organisation, prior to widespread use of NPS. However, when we look at the known mephedrone presentations, only around a third had an ICD-10 code applied that related to the use of a stimulant recreational drug (which would be the most appropriate for mephedrone in the absence of a specific ICD-10 code). This is different to the previous clinical coding study of simulated ARDT presentations, where around 68% applied a primary ICD-10 code of “poisoning by psychostimulants with abuse potential” to a patient with anxiety and palpitations after the use of mephedrone [3]. There have been no changes to the coding practice in the study centres since the cases used in this study, and therefore we would not expect there to have been any improvement in quality of local coding. However, following this study, the proposed ICD-11 coding system was released on the 18th June 2018 [9]. The proposed ICD-11 coding system appears to have incorporated more coding categories related to the use of recreational drugs and novel psychoactive substances, including proposed specific codes for the synthetic cathinones, MDMA and related drugs and the synthetic cannabinoids. These changes may in part help with some of the coding issues that we have identified in this and previous studies, enabling better understanding of the burden of health care utilisation related to the use of a wider range of substances. However, despite the release of the proposed ICD-11 coding system, the expectation is that this will be endorsed by the 72nd World Health Assembly in May 2019 and therefore it is unlikely to be used in routine coding reporting until 2022 onwards.

The European Monitoring Centre for Drugs and Drug Addiction (EMCDDA) through its REITOX national focal point network early warning system collates information on the prevalence of use and harms associated with the use of both classical recreational drugs and novel psychoactive substances [10]. A survey of these national focal points has demonstrated only ~ 50% of countries had any systematic data collection system in place to detect the acute harms of classical recreational drugs and novel psychoactive substances [11]. Those that did largely relied on information from poison information centres and/or data from ICD-10/other clinical coding systems, with limitations on the detail of information provided or the accuracy of detection of cases. The Euro-DEN Plus project enables a detailed data collection currently, in May 2018, within 31 hospitals in 21 European and neighbouring countries, which can provide detailed information to the EMCDDA on recreational drugs/NPS associated with acute toxicity and describe the pattern of acute toxicity seen with different drugs/NPS [4, 8, 12–14]. As such data from the Euro-DEN Plus project now forms part of the annual EMCDDA European Drug Report, adding the existing key indicators around drug detection, drug-related deaths and use of treatment services for drug dependency [15–17]. In the USA, The Toxicology Investigators Consortium (ToxIC) project collects data on cases directly reviewed by medical/clinical toxicologists from a number of hospitals across the USA [18–21]. This has some similarities to the Euro-DEN Plus project, in that a proportion of the cases in the Euro-DEN Plus project have been seen by a medical/clinical toxicologist during their hospital attendance. However, the Euro-DEN Plus project has the advantage of collecting all cases presenting to each individual participating centre irrespective of whether they have been seen at the bedside by a medica or clinical toxicologist. This means cases presenting outside of the time that a medical/clinical toxicologist is on site will also be captured. Additionally, it captures potentially less severe cases who may be discharged directly by the emergency department physicians, rather than needing to involve a medical/clinical toxicologist.

The main limitation of the Euro-DEN Plus dataset is that drug(s) used is based on self-report or clinicians’ interpretation of the presentation for the majority of the cases. The use of self-reported and/or clinical interpretation of drugs involved reflects real-life clinical practice in many hospitals, where the majority of patients do not have analytical confirmation undertaken. A previous sub-group analysis of Euro-DEN Plus presentations with analytical confirmation of drugs used showed there was good correlation between self-reported use of drugs such as heroin, methadone, cocaine, and amphetamines [22]. There was less correlation between cases of NPS-related toxicity, as NPS cannot be detected with the usual drug screening immunoassays, and some substances were not routinely screened for due to limited detection windows in biological matrices (e.g. volatile nitrites and GHB/GBL).

## Conclusion

Currently, the main issue in understanding the burden of acute healthcare utilisation related to the use of recreational drugs and NPS is the lack of easily available locally, regionally, or nationally aggregate data on ED presentations. This is due to the majority of presentations not being coded, inconsistencies in the ICD-10 codes applied to presentations, and lack of ICD-10 codes for the drugs/NPS used. While there are plans to adapt the current ICD coding system [9], further work to develop more automated and easily implementable systems would enable more rapid and complete capture on the acute health burdens associated with drugs and NPS. This would then allow legislative, public health bodies, and emergency/treatment services to understand the overall burden at a national level and regional differences to enable more appropriately targeted treatment service provision and public health interventions to reduce the harms associated with their use.
